# Acute Primary Peritonitis in Cattle

**Published:** 1896-04

**Authors:** J. H. Cramer


Treatment of Acute Primary Peritonitis in Cattle, by J. H. Cramer. Dr. Carsten Hames, in his *Cattle Diseases*, recommends in cases of primary acute peritonitis borax to be given through the mouth, while he makes use of derivantia and Priessnitz's applications (poultices, plasters) externally.

We read in general, even in the Buitenland journals, very little in regard to peritonitis in cattle, and I am free to express the opinion that this disease often occurs as a primary idopathic malady; however named, no reason can be assigned for its appearance. We must either assume that some infectious matter derived from the blood is the cause of the disease, or that a rheumatic influence gives rise to it.

It has occurred to me several times that I have had cattle to treat which ate little or nothing, were very dull, showed a light degree of tympanites, and had a temperature of 40° to 41°, with a rapid pulse, and gave evidence of suffering when the wall of the belly was pressed, so that an ordinary stomach-catarrh could not possibly be the trouble. If we add a typical dashing sound on thrusting them in the left side, caused by the accumulation of fluid in the abdominal cavity, and a usually slow and painful evacuation of manure, these are the principal phenomena which lead me to a diagnosis of peritonitis. In severe cases in which death ensued I found on cutting, for the most part, the typical symptoms of peritonitis. Whenever I have established inflammation of the peritoneum with any degree of certainty I have always treated the patients with large doses of biborax natricus three times daily, 1CO grammes, with some pulvis baccarum juniperi. I have further applied to the wall of the belly a sharp ointment (or plaster), and have had this put on warm.

In one or two days, except in very severe cases, an improvement takes place in the animals so treated, and, as a rule, a full recovery is secured in eight to ten days. In cases of metritis also I have had success with the internal application of borax in large doses.—*Tijdschrift voor Veeartsenijkunde*, January, 1896.

